# Sir St. Clair Thomson's Harveian Lecture

**Published:** 1915-03-20

**Authors:** 


### MODERN METHODS IN RHINO-LARYNGOLOGY.

### Sir St. Clair Thomson's Harveian Lecture.

A few years ago physicians were sceptical when it was suggested that many forms of chronic ill-health had their root and origin in infections of that labyrinth of sinuses and fossæ which are the special field of the nose and throat specialist. To-day, however, it is well understood that microbes such as the pneumococcus, the *Micrococcus catarrhalis*, the *Bacillus influenzæ* and many others commonly present in our surroundings may entrench themselves in remote folds of mucous membrane or cavities adjacent to the nasal passages and there, secure from the ordinary routine procedures of spraying and syringing, manufacture toxins in a steady and persistent manner that sooner or later undermines health. How the fertile brain of Manuel Garcia conceived an idea that was to open up an entirely new branch of surgery, and how the science of rhino-laryngology came into being as the result of that idea, was outlined for the benefit of the members of the Harveian Society last week (March 11) by Sir St. Clair Thomson when delivering the annual Harveian lecture. Taking as his subject "Modern Methods in Rhino-Laryngology," the eminent director of the nose and throat department at King's College Hospital pointed out that the whole specialty, considered in its scientific aspects, really dates from 1855, the year after Garcia first thought of trying to see the vocal cords in the living subject by the aid of a simple mirror such as dentists use in the everyday examination of the mouth. It was, of course, Garcia's explanation of the principles and use of the newly designed laryngoscope, communicated to the Royal Society of London in 1855, that first attracted the serious attention of the medical profession to the new possibilities of investigating the nose and throat thus offered. Since then just sixty years have elapsed, and the developments of rhino-laryngology during that time were considered by Sir St. Clair Thomson as falling into three periods. The first of these lasted up to 1881, and during the quarter of a century that had elapsed between Garcia's discovery and 1881, medical men who had devoted special attention to the nose and throat had made many advances in the art of examining the nasal passages and cavities; but their work had been obstructed by difficulties arising from the extreme sensitiveness of the parts in question. However, 1881 brought with it the discovery of the anæsthetic properties of cocaine, and this placed at the surgeon's disposal a means of getting rid of the sensitiveness of the nasal mucous membrane which had hitherto caused him so much trouble. At the same time hæmorrhage was lessened, and when, at a later date, the ischæmic properties of adrenalin were brought before the notice of the profession, a combination of drugs was at once ready to the hand of the nose and throat expert which not only enabled him to continue his examinations with less difficulty, but to a large extent freed him from the disadvantage of struggling against a vision-obstructing flow of blood. With this important double aid laryngology made great strides, and many operations of the utmost importance to health were devised and became carried out as matters of routine.

Then came another great discovery, largely due to the work of Professor Killian, of Berlin, whose inventive genius brought about the construction of an instrument enabling a direct view of the throat passages to be obtained. This, initiating the third period of development, opened the way for increased facilities for dealing with conditions not only of the larynx, but also of the trachea and bronchi on the one hand, and the œsophagus on the other. It has been during the last ten or fifteen years that direct vision methods have so greatly helped laryngologists and placed in their hands measures which are the most modern of a specialty really in itself modern throughout. Sir St. Clair Thomson exhibited an interesting series of diagrams and $x$-ray photographs clearly showing what a great aid the direct vision apparatus has been to the surgeon in the removal of foreign bodies from the throat. Formerly persons who had the misfortune to inhale a foreign body were only too likely to succumb to its secondary effects or to complications set up by what were described by the Harveian lecturer as the "tempestuous efforts" of the operator to remove them; the danger of a fatal result varying directly with the pointedness of the body inhaled and the depth of its penetration into the lung. One particular diagram shown illustrated how a pin had been successfully removed from one of the secondary bronchi into which it had fallen at a depth of no less than thirteen inches from the teeth. Safety-pins, and tooth-plates with hooked attachment, are not infrequently inhaled, and also sometimes become impacted in the œsophagus, but the difficulties which such mishaps presented quite a few years ago have been overcome by the use of the bronchoscope and œsophagoscope constructed on the principles outlined by Killian. In view of the results that specialists can show to-day there can be no doubt that, where anyone inhales a foreign body, he should without delay be taken to an expert in the use of the direct vision instrument.

Not the least important part of Sir St. Clair Thomson's address was that which dealt with the assistance modern research in rhino-laryngology has given to other departments of surgery apart from the conditions already mentioned. Thus the rhinologist now comes to the aid of the ophthalmologist in cases of lachrymal obstruction, and by making a little window into the nose by the operation known by the somewhat formidable name of dacryo-cystostomy, permits a free outlet for the obstructed secretion. Indeed, the Harveian lecture, which was well attended, must certainly have proved a revelation to any who might still have been inclined to think that nose and throat work consists chiefly in the removal of tonsils and adenoids, or in the application of the cautery and sprays.

